# Transparent polyvinyl-alcohol cryogel as immobilisation matrix for continuous biohydrogen production by phototrophic bacteria

**DOI:** 10.1186/s13068-020-01743-7

**Published:** 2020-06-09

**Authors:** Jan-Pierre du Toit, Robert W. M. Pott

**Affiliations:** Department of Process Engineering, Stellenbosch University, Banghoek Road, Stellenbosch, South Africa

**Keywords:** Polyvinyl-alcohol hydrogel, Photofermentation, Phototrophic bacteria, Bacterial immobilisation, *Rhodopseudomonas palustris*

## Abstract

**Background:**

Phototrophic purple non-sulfur bacteria (PNSB) have gained attention for their ability to produce a valuable clean energy source in the form biohydrogen via photofermentation of a wide variety of organic wastes. For maturation of these phototrophic bioprocesses towards commercial feasibility, development of suitable immobilisation materials is required to allow continuous production from a stable pool of catalytic biomass in which energy is not diverted towards biomass accumulation, and optimal hydrogen production rates are realised. Here, the application of transparent polyvinyl-alcohol (PVA) cryogel beads to immobilisation of *Rhodopseudomonas palustris* for long-term hydrogen production is described. PVA cryogel properties are characterised and demonstrated to be well suited to the purpose of continuous photofermentation. Finally, analysis of the long-term biocompatibility of the material is illustrated.

**Results:**

The addition of glycerol co-solvent induces favourable light transmission properties in normally opaque PVA cryogels, especially well-suited to the near-infrared light requirements of PNSB. Material characterisation showed high mechanical resilience, low resistance to diffusion of substrates and high biocompatibility of the material and immobilisation process. The glycerol co-solvent in transparent cryogels offered additional benefit by reinforcing physical interactions to the extent that only a single freeze–thaw cycle was required to form durable cryogels, extending utility beyond only phototrophic bioprocesses. In contrast, conventional PVA cryogels require multiple cycles which compromise viability of entrapped organisms. Hydrogen production studies of immobilised *Rhodopseudomonas palustris* in batch photobioreactors showed higher specific hydrogen production rates which continued longer than planktonic cultures. Continuous cultivation yielded hydrogen production for at least 67 days from immobilised bacteria, demonstrating the suitability of PVA cryogel immobilisation for long-term phototrophic bioprocesses. Imaged organisms immobilised in cryogels showed a monolithic structure to PVA cryogels, and demonstrated a living, stable, photofermentative population after long-term immobilisation.

**Conclusion:**

Transparent PVA cryogels offer ideal properties as an immobilisation matrix for phototrophic bacteria and present a low-cost photobioreactor technology for the further advancement of biohydrogen from waste as a sustainable energy source, as well as development of alternative photo-bioprocesses exploiting the unique capabilities of purple non-sulfur bacteria.

## Background

Phototrophic purple non-sulfur bacteria (PNSB) have garnered much attention for their ability to produce high-purity hydrogen from organic wastes via photofermentation. In recent decades significant effort has been devoted to developing this potential clean, sustainable energy source to commercial feasibility and in particular overcoming the barrier of enduringly low production rates. In addition to metabolic enhancement, bacterial immobilisation has been identified as key strategy for improving productivity [1]. Hydrogen production occurs independent of growth; thus, the cells can be used as biocatalysts while in stationary phase, with immobilisation facilitating optimal continuous operation of the process. Conventional suspended cell systems require balancing of dilution rates with cell growth to prevent biomass washout [2], thus constraining reactor dynamics to potentially unfavourable parameters. Here cell immobilisation is advantageous, resulting in phase separation of the cells and liquid medium. A stable pool of catalytic biomass is thus maintained within the reactor, irrespective of feed rates, allowing the hydraulic and solids retention times to be precisely tuned for maximal production rate.

Immobilisation offers myriad additional benefits to the bioprocess engineer:

(i) Volumetric biomass loading can be increased over planktonic cells.
(ii) Less metabolic energy needs to be diverted to cell multiplication, resulting in higher product yields.
(iii) Lower risk of genetic drift or reversion.
(iv) Cells are protected from shear stresses and pH, temperature or chemical concentration fluctuations within the reactor [3–5].

While a host of immobilisation matrices have historically been applied to heterotrophic organisms (for an extensive review: [23]) fewer options have been critically investigated for phototrophic bacteria: the key difference being that phototrophic organisms require a high level of transparency for substantial light penetration. Many materials previously studied do not or only partially meet additional key requirements, namely biocompatibility, chemical and mechanical durability for long-term use, and possessing low barriers to diffusion of substrates [12], as illustrated in Table 1.

**Table 1:** Summary of key examples of materials applied to immobilisation of phototrophic bacteria

Matrix material	Organism immobilised	Biocompatibility	Transparency	Diffusion resistance	Mechanical stability	Chemical stability	Refs
Alginate	*Rhodopseudomonas palustris* Photosynthetic consortium *Chlorobium thiosulfatophilum*^*a*^	***Good***	***Good***	***Good***	*Poor*	*Poor*	[6–9]
Agar	*Rhodopseudomonas palustris*	Fair–good	***Good***	Fair	*Poor*	*Poor*	[6, 10, 11]
Polyacrylamide	*Rhodospirillum rubrum*	*Poor*	***Good***	Fair–good	***Good***	***Good***	[12, 13]
Carrageenan	*Rhodopseudomonas capsulata* *Rhodospirillum rubrum* *Rhodopseudomonas palustris*	Fair–good	***Good***	***Good***	*Poor*	*Poor*	[6, 13, 14]
Chitosan	*Rhodobacter sphaeroides*	Fair	Fair	Fair	Fair	Fair	[15]
Boric acid crosslinked PVA	*Rhodopseudomonas palustris* *Rhodobacter sphaeroides*	Poor–fair	***Good***	***Good***	***Good***	***Good***	[16, 17]
PVA cryogel	Various; non-photosynthetic	Fair–good^b^	*Poor*	***Good***	***Good–excellent***^b^	***Excellent***	[18–21]
PVA–glycerol cryogel	*Rhodopseudomonas palustris*	***Good***	***Good***	***Good***	***Excellent***	***Excellent*** [22]	This study

^a^Non-PNSB (phototrophic green sulfur bacterium)

^b^Depending on number of freeze–thaw cycles used to form hydrogel

The early staples of cell immobilisation, agar and alginate, offer good transparency, diffusion properties and biocompatibility, but are exceedingly fragile, and thus unsuitable for long-term industrial use [10, 24]. Alginate is also vulnerable to dissolution in the presence of monovalent cations or chelators [8], placing unrealistic limitations on potential feedstocks. Conversely, robust materials such as polyacrylamide are limited by potentially toxic monomers and adverse polymerisation conditions which compromise cell viability [10].

One underexplored potential immobilisation material is a glycerol co-solvent polyvinyl-alcohol (PVA) cryogel. Cryogelation uses low-temperature cycling to solidify an initially homogeneous liquid polymer solution. Hydrogels formed in this way avoid the use of toxic crosslinking agents, resulting in improved biocompatibility. The extensive physical interactions between hydrophilic polymer chains underlying the gel formation give PVA cryogels a high mechanical strength [25], along with resistance to chemical and enzymatic degradation under typical culture conditions [26]. PVA cryogels exhibit a porous structure [27] which combined with high hydration levels presents minimal barrier to diffusion through the material. As a bulk industrial commodity, PVA is also a very low-cost material for immobilisation at less than 2 USD per kg (Alibaba.com 2019).

PVA-based cryogels have been successfully applied to a variety of fermentation processes over the past few decades, most recently including phenolic degradation [28], and production of ethanol [29]. However, the non-ideal optical properties of conventional cryogels are a barrier to their use for photosynthetic applications. Typical PVA cryogels are functionally opaque, due to the occurrence of pores formed by frozen inclusions, which scatter light. Transparency can be induced by the addition of a polyol co-solvent, such as glycerol [30], which potentially enhances the cryoprotectant effect of PVA itself [31]. While mechanisms for inducing transparency have been described since the late 1980s [32], exploration of transparent cryogels for the immobilisation of phototrophic bacteria has not yet been undertaken. Further, very little physical characterisation of PVA–glycerol cryogel matrices have been conducted to date as specifically pertains to bioprocess use, such as directly examining the mechanical resilience, long-term biocompatibility or productivity of organisms immobilised within the material with the ultimate objective of application to large-scale bioprocesses.

This article demonstrates the successful application of transparent PVA cryogels to photofermentation using the PNSB *Rhodopseudomonas palustris*, a stalwart of biohydrogen research owing to its robustness and extraordinary metabolic versatility [33]. Comprehensive investigation of the material properties showed ideal transparency, high biocompatibility, minimal resistance to diffusion of substrates, as well as excellent mechanical resilience which exceeded that of conventional cryogels after a single freeze–thaw cycle. These characteristics were further validated by batch cultures of cryogel-immobilised *R. palustris* which had higher hydrogen production rates which continued for longer than planktonic cultures, and long-term continuous production in excess of 67 days. Biohydrogen produced from organic waste has great potential as a future green energy source, and optimised immobilisation materials such as transparent PVA cryogels are necessary to coax maximum efficiency from photofermentation processes. Integration of such materials with new photobioreactor designs, along with metabolic engineering of the candidate organisms, has the potential to significantly advance the feasibility of photosynthetic bioprocesses towards commercial scales.

## Results and discussion

### Transparency of cryogels for use with phototrophic organisms

**Fig. 1:**
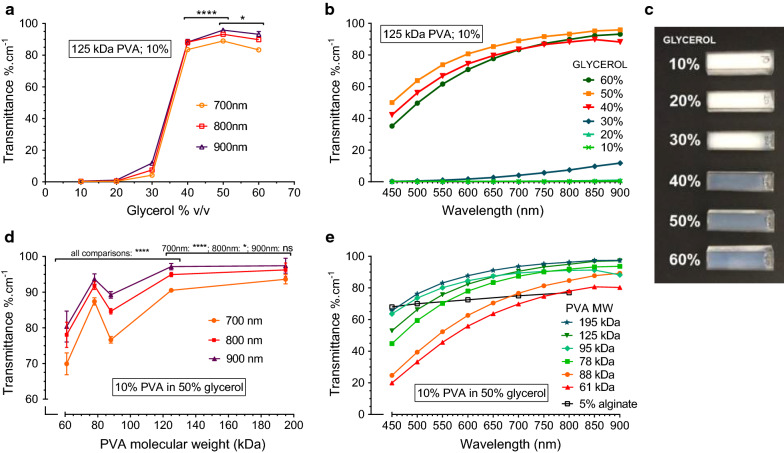
Effect of glycerol co-solvent concentration (**a–c**) and PVA molecular weight (**d, e**) on cryogel transparency. Light transmission quantified in the near-infrared (**a, d**), and overall photosynthetic wavelength ranges (**b, e**) in 10% w/v PVA cryogel after 1 freeze–thaw cycle. PVA samples in cuvettes frozen at −20 °C show changes in opacity relating to glycerol concentration (**c**). Light transmission in 5% Ca-alginate gel films included in panel **e** for comparison from Pereira et al. 2013 [37]. Data shown is the average of 4 samples, with error bars representing SD. Statistical significance for one-way ANOVA with Tukey’s multiple comparison indicated by ****p < 0.0001; *p < 0.05

In order to develop a matrix with optimal light transmittance for photosynthetic organisms, the relationship between the concentration of glycerol in the solvent and the transparency of resultant PVA cryogels was determined (Fig. 1a–c). Light transmittance increases suddenly above 40 vol % glycerol in all wavelength ranges between 450 and 900 nm (Fig. 1b), which correspond closely to photosynthetically active ranges for most microorganisms. Near-infrared (NIR) radiation is particularly important for PNSB relying on bacteriochlorophylls, owing to their evolutionary adaptation to aquatic sediment microenvironments [34]. Transparency in this band between 700 and 900 nm is especially high for solvent systems exceeding 40% glycerol (Fig. 1a), likely due to the reduced attenuation potential of longer wavelengths of light. While light attenuation resulting from concentrations between 40 to 60% was uniformly low, there was a statistically significant optimum concentration of 50%.

As hypothesised by Hyon et al. [32], transparency is induced when phase separation is prevented by depression of the freezing point of the system. This effect was clearly seen in cryogels in the frozen state, with only samples of less than 40% glycerol showing frozen inclusions (Fig. 1c), which in turn is supported by the reported freezing points for glycerol solutions. In pure water-glycerol systems in the range of 40 and 60% v/v glycerol concentration, freezing occurs at −16 °C to −34 °C [35]; low enough to prevent significant phase separation under the cryogelation temperature employed (not accounting for the presence of PVA). At temperatures above −20 °C, rate of phase separation via spinodal decomposition (a form of liquid–liquid phase separation) exceeds the rate of PVA crystallisation, leading to a non-homogeneous material [36]. Tuning the solvent properties of water with increasing amounts of glycerol likely allows widespread crystallisation between PVA chains to occur [30], but concurrently limits crystallite size. Smaller, more numerous PVA crystallites thus minimises scattering of light, resulting in a stable and optimally transparent gel.

Interaction between PVA chains underlies the physical gelation mechanism for cryogels, with longer chains having a higher crosslinking potential. The effect of PVA molecular weight on transparency was thus investigated (Fig. 1d, e). All molecular weights of PVA tested showed good light transmission, particularly in the critical near-infrared wavelength range between 700 and 900 nm (Fig. 1d, e). Light transmission in this range was typically superior to 5% calcium alginate hydrogels [37], the most widely studied immobilisation material for photosynthetic applications due to its transparency [8].

A trend of transparency increasing with PVA molecular weight was seen (Fig. 1e), but was slightly obfuscated by the relatively low specification in terms of hydrolysis of the PVA used (≥ 98%) and the complex interplay between the factors affecting the physical gelation process and the resultant optical properties. With increasing degree of hydrolysis, the overall hydrogen bonding capacity of the polymer increases significantly. Between 95 and 100% hydrolysis, hydrogen bonding capacity increases by approximately 30%, leading to preferential interactions between PVA chains over those with water [38]. Subtly lower degrees of hydrolysis would appreciably reduce the rate of crystallite formation, thus shifting the equilibrium towards phase separation in advance of extensive gelation and subsequently impacting material homogeneity.

While ideal transparency is important for photosynthetic bioprocesses, using widely available inexpensive industrial grades of PVA would not greatly impact the optical properties of the gel or its suitability as an immobilisation matrix. PVA with both a higher degree of hydrolysis and molecular weight is preferable for optimal transparency, as long as solution viscosity remains practical for the immobilisation procedure. High PVA molecular weights result in solutions with commensurately high viscosity, which complicates the dripping procedure used in this study to form cryogel beads. A balance between such practical considerations and ideal optical properties should be found for the particular protocol employed, and recommendations will be discussed.

In the NIR wavelength range, all except the lowest of the PVA molecular weights tested exhibited good transparency for use with photosynthetic bacteria (Fig. 1e). Since the ultimate thickness of the matrix would be on the order of a few millimetres to avoid limiting diffusion of solutes within the material [12], the matrix would offer insignificant impedance to incident light in contrast to other materials which are functionally opaque.

### Mechanical properties of transparent cryogels for bioprocess use

**Fig. 2:**
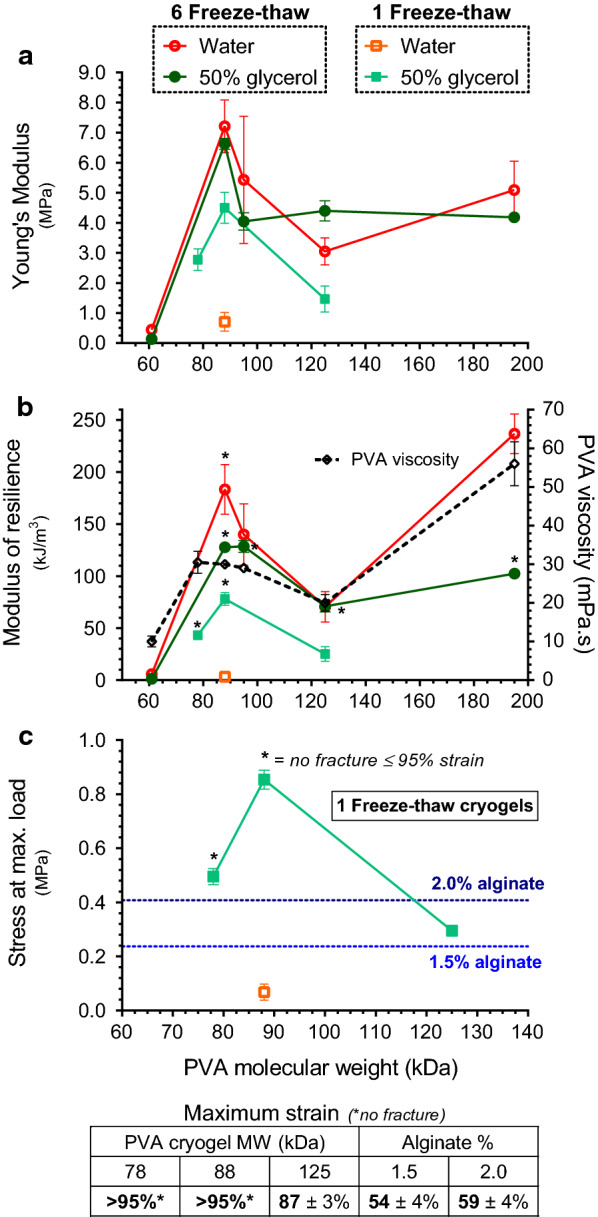
Compressive characteristics of cryogels comprised of PVA with varying molecular weights. Cryogels formed from 10% w/v PVA solution in either 50% v/v glycerol (transparent) or pure water (conventional). 4–8 samples were used to determine average Young’s modulus (compressive modulus, **a**), modulus of resilience (**b**) and load under maximum stress (**c**) after the indicated number of freeze–thaw cycles (FT). Standard dynamic viscosities (4% solution, 20 °C) from manufacturer’s specifications for PVA samples tested show correlation to cryogel resilience (**b**). Reference fracture stresses and strains indicated for 1.5 [44] and 2% alginate hydrogels [45] (**c)**. Error bars represent SD. Data not shown for samples which did not form stable cryogels under specified conditions (1FT, transparent 95 and 195 kDa; 6FT: 78 kDa were not investigated)

Industrial application of immobilisation matrices will require high material resilience to withstand shear stresses and abrasion present in bioreactor environments, both for long-term production and reuse of the biocatalyst. As cryogel structure and integrity is dependent on the number freeze–thaw cycles it is subjected to, the compressive strength characteristics of conventional (water solvent) and transparent (50% glycerol co-solvent) cryogels were determined after single and multiple freeze–thaw cycles (Fig. 2), as an indication of how well these materials will fare in mechanically challenging bioreactor environments.

Values of Young’s modulus, a measure of the elastic compressibility or stiffness of the material, demonstrated a very elastic and compliant material with the transparent cryogels exhibiting marginally lower levels of stiffness (Fig. 2a) and thus resilience (Fig. 2b) than conventional cryogels, but nonetheless remained high. While all PVA molecular weights in both solvent systems formed stable cryogels after 6 freeze–thaw cycles, forming stable conventional PVA cryogels after a single freeze–thaw cycle was not feasible, and only the 88-kDa samples formed very diffuse gels with low fracture stresses of less than 70 kPa (Fig. 2c).

Higher molecular weights of PVA would be expected to increase cryogel strength due to the higher propensity for interactions between polymer chains as their average length increases. However, there was no clear trend of resilience increasing with molecular weight (Fig. 2b). PVA gelation is a complex, multifactorial process depending on the intrinsic properties of the sample used. Factors include not only molecular weight and degree of hydrolysis as discussed previously, but also the specific polymer stereoregularity, polydispersity of chain length and both the thermal history and degree of dissolution of the PVA in solution [39]. As reported by Lozinsky et al., increasing polymer chain length also seems to be a hindrance to efficient interchain interactions, since the concomitantly increasing viscosity in solutions of higher molecular weight PVA constrains mobility during the gelation process [40]. Similarly, higher cryogel strength was reported for 86-kDa PVA samples compared to 115 and 66-kDa, closely replicating the local optimum seen here at 88 kDa. Modifying PVA concentration was thus seen as a better tool for controlling cryogel properties in place of increasing molecular weight beyond this balancing point for intermolecular interaction propensity. In turn, comparison of manufacturer specifications for the PVA samples used showed a degree of correlation between standardised dynamic viscosity values and the resilience of the cryogel formed (Fig. 2b). While these single-point values do not reflect the intrinsic viscosity properties of the PVA sample, which would provide better prediction of material properties via more comprehensive rheological characterisation [41], they provide a rough guide for selection of suitable PVA samples. Standard viscosity values in the range of 30 mPa.s were shown to offer good cryogel strength while maintaining sufficient solution fluidity to facilitate the formation of cryogel beads via the dripping method used in this study.

As in the case of inducing transparency, the presence of the glycerol co-solvent is again advantageous for cryogel integrity. During the gelation process, lowering temperature constrains molecular mobility and facilitates crystallite formation as long as the system remains in solution [42]. At the point of freezing, further crystallite formation is inhibited and thus the extent of gelation is dependent on cooling rate up to the freezing point. Depression of the freezing point of solutions containing glycerol allows optimal crystallite formation independent of cooling rate, since the freezing point is reached more slowly (or not at all). In the absence of sophisticated control of freezing rate, conventional PVA–water solutions thus require multiple freeze–thaw cycles to allow widespread crystallite formation to form stable gels.

Synergistically, the kosmotropic effect of glycerol further reinforces the gel structure by favouring physical bonding between polymer chains over polymer–solvent interactions, significantly increasing both gel strength and thermostability [30, 43]. Here the transparent cryogels offer an advantage in terms of simplicity of immobilisation protocol and minimising deleterious impact on entrapped cells. While conventional cryogels exhibited slightly higher resilience and thus robustness to deformation than transparent gels after multiple freeze–thaw cycles (Fig. 2b), yielding a strong material after a single freeze–thaw cycle is a significant advantage for microbial immobilisation. Successive cycles of varying temperature are detrimental to living cells, so minimising such adverse process conditions to a single freeze–thaw cycle would best preserve the viability of entrapped organisms as explored further in Sect. “Assessment of PVA immobilisation procedure on cell viability”. We thus focused exclusively on the single freeze–thaw cycle transparent cryogels for further characterisation. These cryogels compared favourably to alginate gels (Fig. 2c) since both fracture stress and strains reported for 1.5 [44] and 2% alginate [45] were much lower than those resisted by single freeze–thaw transparent cryogels. At 95% strain, 88-kDa cryogels withstood at least double the fracture stress of 2% alginate which failed at only ~ 60% deformation.

Indeed, the resilience shown reflects only the bare minimum values, as the majority of samples did not actually fracture below the very high maximum strain of 95% investigated here (Fig. 2c). Single-freeze–thaw cycle 88-kDa transparent cryogels withstood in excess of 10 such compressions with no significant change in Young’s modulus or maximum stress (data not shown). These conditions exceed forces likely present under even very vigorously mixed bioprocess conditions, and no discernible degradation of the PVA beads was seen after multiple-month use under constant robust agitation.

Transparent cryogels are thus demonstrably extremely robust materials, outperforming conventional cryogels by realising these properties after single freeze–thaw cycles, and thus showing promise for even non-phototrophic bioprocesses where maximum viability of entrapped entities is required or desirable with the additional benefit of a faster, simpler procedure.

### Morphological differences between conventional and transparent cryogels

A hallmark of conventional PVA cryogels is a macroporous structure [27], which in turn underlies the opacity of the material. Transparent cryogels are conversely less porous in nature, allowing high transmittance of photosynthetically active wavelengths of light. Confocal microscopy of conventional and transparent cryogels negatively stained with fluorescein further support the hypothesis that transparency is due to the formation of a homogeneous, monolithic, material. Imaging of gels in such a way in the native hydrated state avoids sample preparation artefacts introduced by alternative techniques such as scanning electron microscopy (SEM), which tends to collapse gel structure during the requisite freeze-drying process [46] and obfuscated the subtle morphological differences between the cryogel types studied here.

**Fig. 3:**
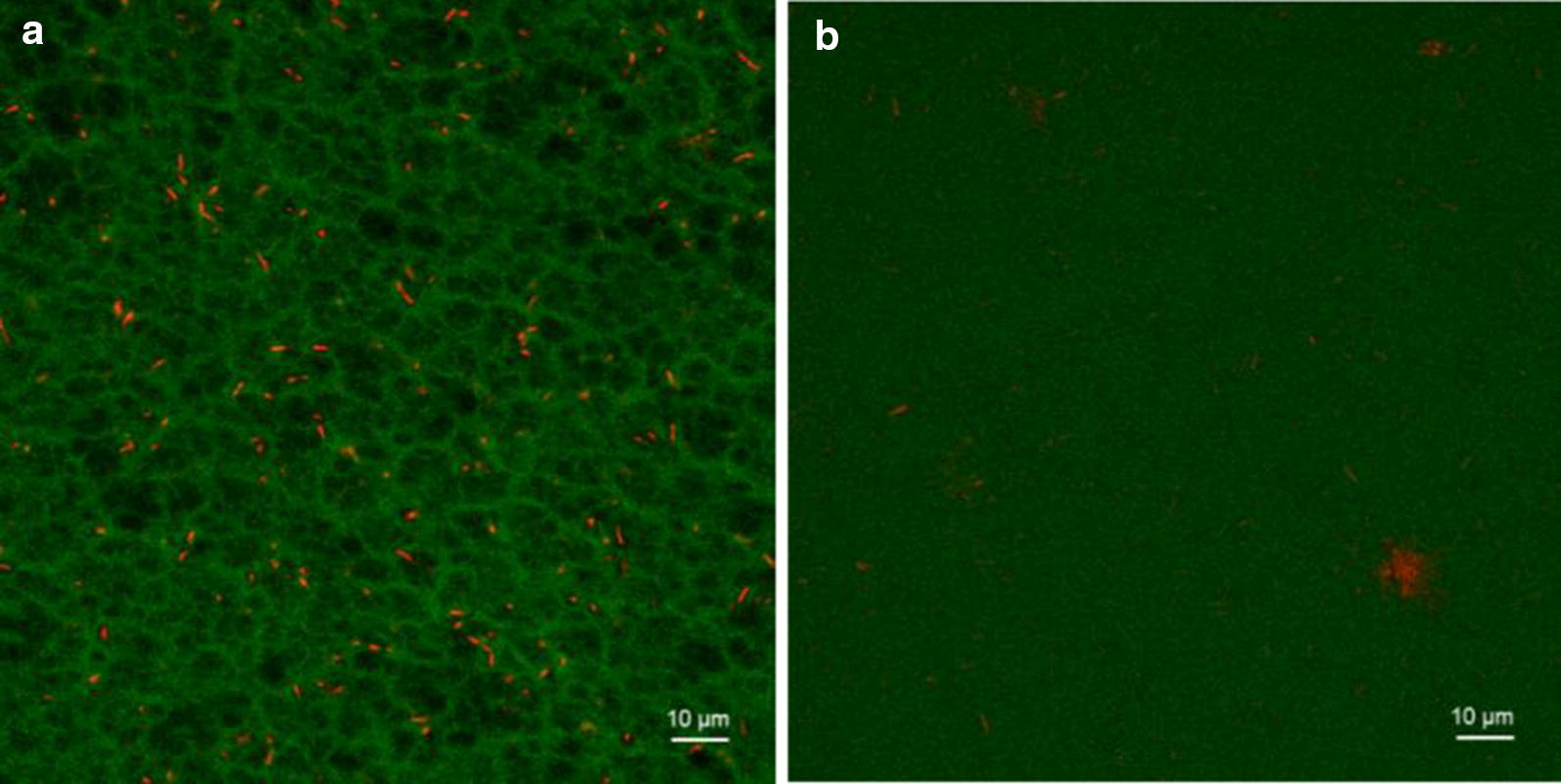
Confocal laser scanning microscopy (CLSM) visualisation of pore structure in different PVA cryogels. Cryogels formed from 10% w/v PVA (88 kDa) in water (**a**—conventional) and 50% glycerol (**b**—transparent), with immobilised *R. palustris*. *R. palustris* autofluorescence is represented in red; negative staining with fluorescein shows lower densities of cryogel in increased green fluorescence intensity. Optical sections taken 30–40 µm from gel surface. Images acquired and processed identically, with contrast increased for visibility

In Fig. 3a, porosity can be inferred from the relative concentration of the staining agent, while Fig. 3b shows a much more uniform (and therefore non-porous, and monolithic) staining.

The conventional gel shows porosity clearly resulting from phase separation, with high-density PVA crystallites surrounded by low-density features running through the gel represented by higher fluorescein fluorescence (Fig. 3a). This morphology corresponds well to previous studies of conventional PVA cryogels [25]. In contrast, the transparent cryogel shows uniform staining (Fig. 3b) indicative of homogeneous gel density, or at least microporosity not visible using optical methods.

The resolution limit of the confocal microscopy technique used is around 0.5 µm; any potential pores present smaller than this would still facilitate mass transport [47]. Conversely, in the highly hydrated hydrogel matrix comprising around 90% water, porosity may not confer a major advantage in terms of ease of diffusion.

### Diffusive properties of cryogels

**Fig. 4:**
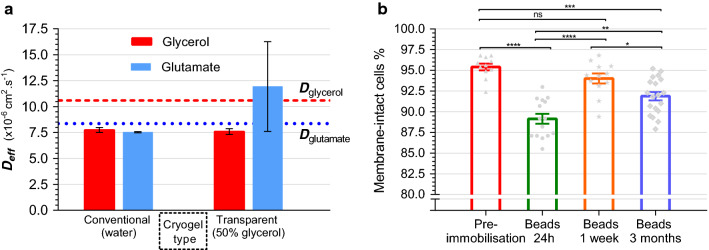
Characterisation of biologically relevant properties of PVA cryogels for bioprocess applications. Diffusion of glycerol and glutamate (candidate substrate molecules) in conventional and transparent PVA cryogels quantified at 35 °C, in terms of the effective diffusion coefficient, *Deff* (**a**). Reference aqueous diffusion coefficients, *D*, are indicated by labelled dotted lines. Error bars represent SD of 2 replicates. Biocompatibility of cryogels was determined by comparing cell viability before and after immobilisation in PVA cryogel beads under continuous cultivation (**b**). Cell-membrane integrity is used as proxy for cell viability, expressed as percentage intact cells:total cells as measured by LIVE/DEAD staining and CLSM. Error bars represent SEM for minimum of 3 samples with 3–4 images each; datapoints shown as grey symbols. Statistical significance for one-way ANOVA with Tukey’s multiple comparison indicated by ****p < 0.0001; ***p < 0.001; **p < 0.01; *p < 0.05; *ns* not significant

The reduction in porosity in transparent cryogels may affect the diffusion characteristics of the material, since at first impression macroporous gels may seem to be at advantage in terms of permeability. However, porosity is not always directly correlated to diffusivity since the degree of tortuosity of the structure significantly affects mass transport [48]; and indeed, in hydrogels diffusion through the gel is not nearly as limited as diffusion through other solids in which porosity is an important factor (such as activated carbons). In order to determine the contribution of macroporosity to the apparent diffusivity in cryogels, diffusion studies were performed by measuring effusion of glycerol and glutamate from cryogel cubes (Fig. 4a). These substrates are representative carbon and nitrogen sources commonly used for *R. palustris* cultivation and present a range of solute molecular weights at around 92 and 146 kDa, respectively. Glycerol is an attractive substrate for biohydrogen production, and is abundant due to the worldwide glut as a waste product of biodiesel manufacture [49].

Effective diffusion coefficients of both glycerol and glutamate in Fig. 4a showed no significant difference between conventional and transparent cryogels, indicating an apparent lack of influence of gel macroporosity. The measured diffusivity of glycerol is also in good agreement with published values for aqueous diffusion coefficients in infinite sink systems, at 10.6 × 10^−6^ cm^2^.s^−1^ [50], which represents a high relative diffusivity of 72% (expressed as the ratio of effective to standard aqueous diffusion coefficients). Glutamate diffusion (Fig. 4a) followed a similar trend of close correspondence to aqueous diffusion rates of 8.36 × 10^−6^ cm^2^.s^−1^, albeit measured at 10 °C lower and under infinite sink conditions [51]. Alginate, the mainstay of immobilisation matrices for its excellent permeability, has a relative diffusivity of 86% at 5% polymer concentration [52]. A similar relationship was seen for a range of common carbon sources in 2% alginate hydrogels, with an average relative diffusivity of around 85% [53]. Alginate thus offers only modestly higher permeability than the 10% PVA cryogels characterised here. Indeed, it would seem that the highly hydrated nature of the material presents minimal obstacle to passive mass transport through the material, as supported by studies of glucose diffusion in conventional cryogels which showed very similar relative diffusion rates [54].This permeability is likely further enhanced by the swelling of the material upon removal of the glycerol co-solvent and the expansion of the gel matrix following cryogelation [26]. Despite possessing differing molecular weights, effective diffusion coefficients for glycerol and glutamate were not significantly different (Fig. 4a). Hydrodynamic radius of the diffusing molecule is more relevant to its mobility than simple molecular weight, and the structures of glycerol and glutamate suggest little difference in this respect although this could not be confirmed due to lack of reported experimental values. PVA cryogels are highly hydrophilic due to the abundance of hydroxyl groups projecting from the polymer backbone, thus the degree of hydrophilicity of the substrate molecule would likely contribute additional influence on diffusion rates through the matrix, perhaps warranting further investigation beyond the scope of the tests performed here.

From a process perspective, the high relative diffusion rates in transparent cryogels would likely result in minimal impact on substrate availability. For reference the aquaglyceroporin GlpF, responsible for glycerol transport in *E. coli,* with homologs in *R. palustris* [55], has a maximum predicted diffusion rate of 3.0 × 10^−6^ cm^2^.s^−1^ [56]; or roughly half the diffusion rate through the hydrogel matrix, making transport into the cell slower than transport to the immobilised cell. PVA cryogels thus offer attractive mass transport properties for bioprocess use, at a similar scale as ideally permeable immobilisation materials, with the added advantages of excellent robustness and good transparency.

### Assessment of PVA immobilisation procedure on cell viability

**Fig. 5:**
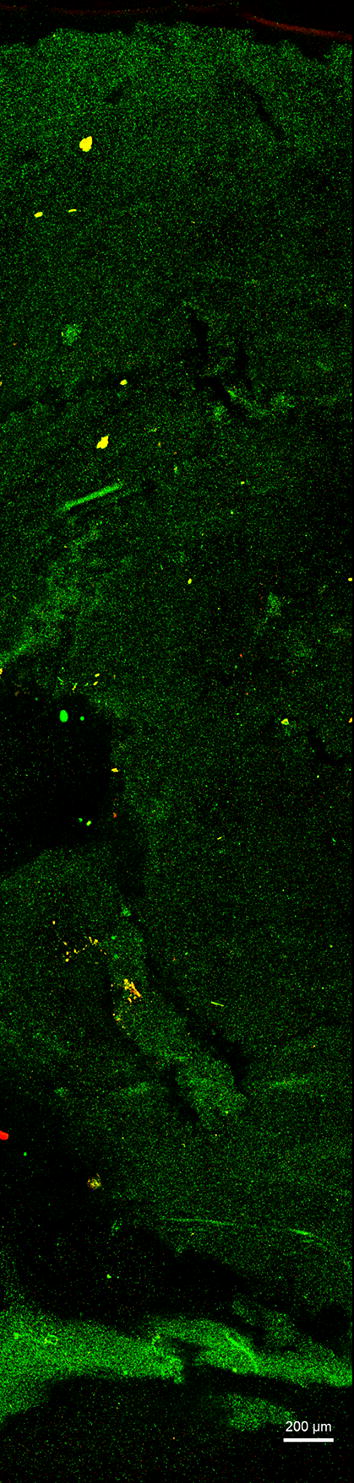
CLSM micrograph of LIVE/DEAD stained *R. palustris* entrapped in PVA cryogel bead following 24 h cultivation in minimal media under illumination. Image presents entire cross-section through centre of a single PVA cryogel bead. Membrane-intact cells are represented by green fluorescence (Syto9) and membrane-compromised cells in red (propidium iodide). Yellow inclusions are non-bacterial debris as artefact of immobilisation process. Image contrast enhanced for visibility equally across all channels

Exposure to low temperature has the potential to adversely affect cell viability, since formation of ice crystals can rupture cell membranes resulting in cell death. Diminished catalytic activity associated with loss of viability of entrapped bacteria is therefore a possible drawback for an immobilisation matrix formed by cryogelation. The proportions of cells with intact and compromised cell membranes was thus determined before and after immobilisation, shown in Fig. 4b. A ~ 6% drop in membrane integrity to 89% was seen in immobilised bacteria following 24-h cultivation, from a maximum of 95% pre-immobilisation, suggesting a negligible impact on cell viability. A full cross-section through a PVA bead in Fig. 5 further demonstrates even distribution of intact viable cells throughout the material, with slight non-homogeneity resulting from manual mixing of the cell suspension into the PVA solution prior to immobilisation.

Since cryoinjury occurs from ice formation both within and outside the cell, cryoprotectants are typically used to prevent damaging ice formation when cells are frozen for storage [57], mediated by their colligative properties depressing the freezing point and thus reducing the propensity for ice crystal formation throughout the system. This echoes the role of the glycerol co-solvent in inducing cryogel transparency; hence, glycerol serves double duty by additionally reducing cell damage during the immobilisation process. Indeed, this protective effect extended further to the integrity of the cryogel beads formed by the immobilisation procedure employed here. Conventional PVA–water solutions fractured violently upon freezing in liquid nitrogen, likely due to large shear stresses in the material occurring during rapid crystallisation, further compromising bacterial integrity and requiring slower alternative freezing techniques [18].

The rapid freezing induced by dripping into liquid nitrogen, aided by the presence of glycerol, results in vitrification of the PVA beads and the cells contained within them. This glassy, amorphous state results from the rapid increase in viscosity precluding crystallisation, further limiting cryoinjury to cells [58]. Upon warming, this vitreous state of the cells is vulnerable to crystal formation as temperatures increase and molecular mobility returns. PVA has shown promise as an alternative cryoprotectant due to its ability to inhibit ice recrystallisation at low concentrations [59], and this property potentially acts in synergy with the glycerol co-solvent to enhance the biocompatibility of the PVA cryogelation process.

Following cultivation, the proportion of membrane-intact entrapped cells rebounded to 94% after 1 week (Fig. 4b), suggesting that the majority of membrane damage resulting from the freezing process was not fatal, and that cells were able to recover completely to pre-immobilisation levels. Indeed, the disruption in membrane integrity represented by positive staining with propidium iodide (PI) does not necessarily equate to cell death, leading to underestimations of true overall cell viability [60]. Here, the recuperation after membrane damage seems to support this observation. Notably, the recovery occurred while cultivated in minimal medium and without a nitrogen source, and entrapped cells showed only a 2% decrease in observable viability after 3 months of continuous culture under 24 h illumination. These observations further confirm both the resilience of *R. palustris* [33], and the biocompatibility of both the immobilisation process and the PVA cryogel material itself, making this technique well-suited for long-term photofermentation.

### Phototrophic biohydrogen production by PVA cryogel-entrapped *R. palustris*

**Fig. 6:**
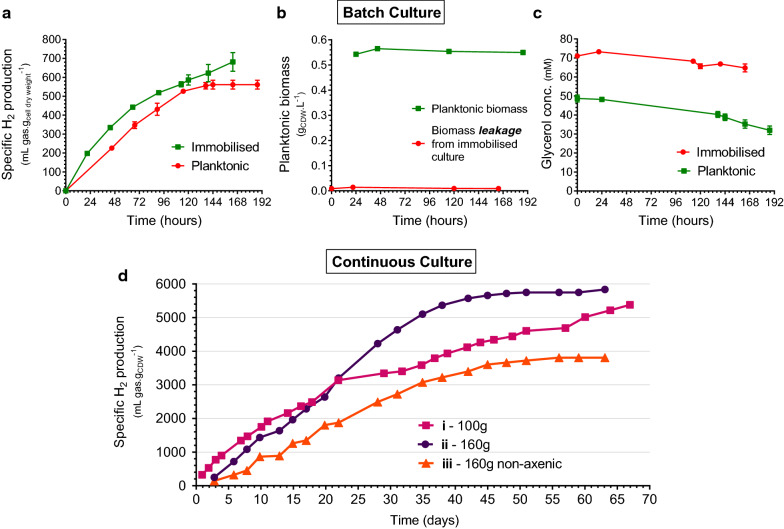
Phototrophic hydrogen production by non-growing PVA-immobilised *R. palustris*. Nitrogen-free minimal media with 50 mM glycerol used for cultivation under illumination in test bioreactors in batch mode (**a–c**), and continuous mode at a dilution rate of 0.01 h^−1^ (**d**). Hydrogen volume was normalised to biomass in reactor, reported as specific hydrogen production (**a, d**). Equal biomass was loaded into planktonic and immobilised reactors at the start of the experiment and concentration was tracked to confirm lack of growth and to monitor cell leakage from PVA beads (**b**). Glycerol concentration was measured to track carbon consumption (**c**). Error bars represent SD for minimum 3 replicate reactors. For long-term continuous photofermentation (**d**), varying quantities of beads were loaded into single reactors under both axenic (**i**: 100 g, **ii**: 160 g) and non-axenic conditions (**iii**: 160 g)

To verify the utility of the transparent PVA cryogel as an immobilisation matrix for photosynthetic bacteria, biohydrogen production using immobilised and planktonic cultures of *R. palustris* were compared in test-scale bioreactors in batch mode with identical biomass loading (Fig. 6a). Nitrogen-free media was used to maintain cells in non-growing state to allow comparison of bacterial activity from a stable, directly comparable biomass complement free from the additional energy sink of cell replication. The non-growing state of planktonic cultures was confirmed by monitoring biomass concentration, along with levels of biomass leakage from the PVA beads (Fig. 6b). Constant, negligible concentrations of planktonic biomass in the immobilised reactors confirmed robust and stable entrapment of cells within the matrix, an advantage over alternative immobilisation materials with large pores with consequently lower stability which are prone to biomass loss [61]. Immobilised *R. palustris* showed a modestly higher total hydrogen productivity compared to control cultures, suggesting a lack of any detrimental effect of the cryogelation procedure on cell viability and activity (Fig. 6a). This is consistent with the analysis of post-immobilisation membrane integrity in the previous section, confirming that no discernible metabolic damage was inflicted by cryogelation. In addition, immobilised cultures displayed a higher maximum specific hydrogen production rate at 8.0 mL.g^−1^.h^−1^, compared to 5.1 mL.g^−1^.h^−1^ (Fig. 6a; *p* < 0.0001). Analysis of glycerol showed a higher initial concentration for immobilised cultures, likely from residual efflux of glycerol co-solvent from the PVA cryogel (Fig. 6c). A short 24-h washing step was used to equilibrate the cryogel beads before the start of the batch culture to allow accurate comparison to planktonic cultures (see Methods Sect. “*R. palustris* immobilisation”), leading to a higher initial glycerol concentration due to residual glycerol remaining from the immobilisation procedure. The rate of glycerol consumption following the initial 24 h of batch culture, however, was indistinguishable statistically between the groups, suggesting equilibration of glycerol concentration within and outside the beads and thus equivalent accessibility of glycerol substrate to both culture types. This lack of apparent mass transport limitation to immobilised cells (Fig. 6c) was supported by the glycerol diffusion experiments (Fig. 4a). For industrial applications, the presence of the glycerol co-solvent as a fermentable substrate within the immobilisation matrix would indeed likely be advantageous to foster maximal post-freezing recovery of cells. Extensive washing of the beads would therefore not be required before use as in the case of alternative transparent cryogels employing toxic DMSO as the co-solvent [36].

Hydrogen production from immobilised cells continued for a longer period than planktonic cultures (Fig. 6a) which all but ceased after 6 days perhaps due to accumulation of deleterious waste materials, although the exact cause was not determined. Increased productivity of immobilised PNSB in terms of both rate and duration has been widely reported, and forms part of the rationale for this work. Vincenzini et al. described sustained hydrogen productivity from organic acids continuing for at least 60 h in agar-immobilised cultures, compared to 25 h in suspension [11]. Fiβler et al. similarly reported that immobilised *R. palustris* produced hydrogen from toxic aromatic acids for longer periods than planktonic cultures [6], suggesting a potential protective effect against adverse conditions offered by immobilisation. Further supporting the suitability of the cryogel matrix, entrapment within the PVA beads shows no evidence of practical limitation in terms of availability of light to drive the bioconversion process. This reinforces the observations made from the diffusion studies, and the favourable light transmission properties of the material. Although substrate diffusion and light penetration are not likely to be the rate limiting steps in the hydrogen production pathway [62], the suitability of transparent PVA cryogels for photofermentation is further established.

Since long-term productivity is a key goal for immobilised bacteria, PVA-entrapped *R. palustris* was subjected to continuous non-growing cultivation in three test bioreactors fed with 50 mM glycerol in nitrogen-free media at a constant dilution rate of 0.01 h^−1^, with varying quantities of PVA beads loaded and under axenic and non-axenic conditions, as seen in Fig. 6d. Hydrogen production was sustained for at least 45 days in all three reactors. The longest sustained production was seen for the lowest bead loading of 100 g (Fig. 6d, i), which produced hydrogen at an initial rate of 127 decreasing to 53 mL $g_{CDW}^{ - 1}$day^−1^. Hydrogen evolution continued for at least 67 days, at which point the supply of sterile media was exhausted, representing minimum sustained productivity of over 2 months from a stable pool of catalytic biomass. Higher bead loading of 160 g resulted in a higher specific production rate of 154 mL $g_{CDW}^{ - 1}$ day^−1^being maintained over 34 days (Fig. 6d, ii), followed by tapering off in hydrogen evolution. Under non-axenic conditions, average production rate decreased to 94 mL $g_{CDW}^{ - 1}$ day^−1^ (Fig. 6d, iii), likely due to competition from contaminating organisms for the available carbon. The presence of these non-PNSB organisms was detected as a white cloudiness in the culture medium and were speculated to be *Bacillus* species, which are common culture contaminants capable of glycerol utilisation. However, production in this reactor continued for a similar time period, indicating a similar performance duration for commercial-scale processes which will likely use non-sterile feedstocks to reduce input costs with the goal of reaching economic feasibility.

Interestingly, the viability of entrapped bacteria after 3 months of continuous culture remained high at 92% intact cells (Fig. 4b); a drop of 3% from pre-immobilisation. Cessation of hydrogen production was therefore seemingly not due to cell death, but rather another metabolic cause, perhaps relating to the absence of a nitrogen source for continued enzyme production, or the low dilution rate used which may have caused nutrient limitation due to the long hydraulic retention time (HRT). While these production rates are average in context of benchmark values of up to 45 mL g^−1^h^−1^ for batch culture achieved in literature [63], they do compare favourably to alginate immobilisation studies where maximum production rates of 3.7 mL g^−1^ h^−1^ were reported [6] The simple test bioreactor system used here is non-optimised in terms of light penetration, so does not likely reflect limitations on the part of the cryogel immobilisation matrix itself, but rather the bioreactor configuration, which is the subject of ongoing improvement. HRT also has a significant influence on hydrogen production rate [64], and since the long 100-h HRT investigated here was chosen to maximise glycerol conversion and the feasible duration of the experiment, there is much potential for improvement at the expense of lower substrate conversion. The long-term productivity facilitated by immobilisation in PVA cryogels, combined with the excellent resilience of the material, advance the candidacy of these materials for large-scale photo-bioprocess use.

## Conclusion

In summary, this work posits transparent PVA cryogels as an ideally suited immobilisation material for phototrophic bacteria. The addition of 50% glycerol as a co-solvent induces optimal transparency in normally opaque cryogels by reducing ice crystal formation and fostering gel homogeneity, with an added cryoprotective effect which preserves the viability of entrapped bacteria, visualised here in situ. The material displays high mechanical strength and durability which is attractive for industrial-scale bioprocesses. Due to the reinforcing effect of the glycerol co-solvent these properties are achieved after a single freeze–thaw cycle, presenting a distinct advantage over conventional cryogels which require multiple cycles and extending applicability beyond phototrophic processes. Diffusion studies show minimal barrier to movement of common substrates such as glycerol and glutamate through the matrix, despite the abolition of macropores in the transparent cryogel. Biohydrogen production by photofermentation with entrapped *R. palustris* continued for longer periods than equivalent planktonic cultures, and continuous cultivation yielded productivity for at least 67 days, demonstrating the long-term applications of PVA cryogel immobilisation. The combination of these positive characteristics and the low cost of materials makes transparent PVA cryogels attractive for the advancement of industrial-scale biohydrogen production, as well as new photo-bioprocesses exploiting the unique capabilities of phototrophic bacteria.

## Materials and methods

All reagents used were of minimum reagent grade and purchased from Sigma-Aldrich unless otherwise stated. Atactic polyvinyl-alcohol (PVA) with approximate molecular weights ranging between 61 and 195 kDa and at least 98% hydrolysis (except 96% for the 95 kDa PVA) were used: Aldrich, Mowiol^®^: 61 kDa (10–98), 125 kDa (20–98) and 195 kDa (56–98); Scientific Polymer Products (NY, USA): 88 kDa (Cat# 362) and 95 kDa (#351); Polysciences Inc. (PA, USA): 78 kDa (Cat# 15,130). 10% w/v aqueous solutions were prepared in either deionised water or 50% v/v glycerol by heating to ~ 95 °C under magnetic stirring for 8 h in capped reagent bottles to minimise evaporation.

### Cryogel sample preparation

Hot (~ 60 °C) PVA solutions were poured into either cubic silicone molds (20 x 20 x 19 mm) or 3 mL polystyrene cuvettes and subjected to the indicated number of freeze–thaw cycles to form cryogels: cooling to −20 °C for 12 h, followed by thawing at ambient temperature for 4 h. Cubes were washed 5 times in excess volume deionised water over at least 10 days to hydrate the cryogel and remove glycerol co-solvent.

### Mechanical testing

A benchtop universal testing machine (Lloyd instruments) was used to determine the Young’s modulus (in the linear viscoelastic region) and yield stress of cryogel cubes at controlled room temperature (~ 20 °C) under a constant unconstrained uniaxial compression rate of 20 mm.min^−1^ (*n* ≥ 4). The modulus of resilience was calculated using the formula:

$$E_{r} \, = \,\frac{{Yield\,stress^{2} }}{{2\left( {Young's\,Modulus} \right)}}.$$

In samples which did not rupture below 95% strain, maximum stress was used as surrogate for yield stress.

### Optical characterisation

The optical density of swelled PVA samples in polystyrene cuvettes (*n* = 4) with path length of 1 cm was measured in scanning mode over a wavelength range of 450 to 900 nm in a UV–Vis spectrophotometer (Varian Cary 1E).

### Diffusive properties

PVA cryogel cubes (~ 6 cm^3^) were equilibrated to desired substrate concentration with 5 changes of solution over at least 5 days (Glycerol: 120 mM, monosodium glutamate: 60 mM; high purity in deionised water). Cubes were pre-warmed to 35 °C in the equilibration solution, blotted dry, measured to 0.1 mm accuracy and added to 40 mL distilled water in a sealed 100 mL temperature-controlled reactor at 35 °C with magnetic stirring at 250 RPM. 200 µL samples were taken at intervals to measure effusion of substrate from the cubes. The diffusion coefficient was calculated by solving for the diffusion inside and flux out of the cube, where the cube was treated as a sphere with a diameter equal to the Sauter mean diameter of the cube. The diffusion coefficient was then regressed to the time series measurements of bulk concentration. Supplementary method details with sample calculations can be found in Additional file 1.

### Bacteria and culture methods

*Rhodopseudomonas palustris* strain NCIB 11774 was obtained from ATCC (ATCC^®^ 17007™) and grown in modified Rhodospirillacea minimal medium consisting of (L^−1^): 0.57 g KH_2_PO_4_, 1.86 g K_2_HPO_4_, 0.4 g NaCl, 0.2 g yeast extract, 0.25 g Na_2_S_2_O_3_.5H_2_O, 0.2 g MgSO_4_.7H_2_O, 0.05 g CaCl_2_.2H_2_O, 0.005 g Ferric citrate, 0.002 g para-aminobenzoic acid. Growth medium was adjusted to pH 7.2 before autoclaving, with subsequent addition of sterile glycerol (to 50 mM), glutamate (to 10 mM) and 1 mL.L^−1^ trace element solution [49]. Nitrogen-free media used for non-growing hydrogen production studies omitted yeast extract and glutamate. For rapid preparation of immobilisation biomass, Van Niel’s Yeast Medium with 50 mM glycerol was used (VNG medium; 1 g K_2_HPO_4_, 0.5 g MgSO_4_, 10 g yeast extract,.L^−1^). Cultures were streaked onto VNG agar plates to confirm absence of contaminating organisms before further use.

### Photobioreactor setup

Cultures were grown in 500-mL bioreactors comprised of borosilicate reagent bottles (Simax) and polypropylene lids fitted with gastight stainless-steel liquid and gas sampling tubes; sterilised by autoclave. Anaerobic and N_2_-free conditions were induced by sparging cultures with argon gas through a 0.2-µm PTFE filter for 10 min. Agitation was provided by magnetic stirring at ± 200 RPM with 50-mm PTFE-coated stirrer bars and illumination by 100 W tungsten-filament incandescent lightbulbs. Irradiance intensity was calibrated to 200 W.m^−2^ (± 20) at the internal surface of the bioreactors in the wavelengths between 500 and 1100 nm, corresponding to the range utilised by *R. palustris*, using a compact spectrometer with cosine correcting probe (RGB photonics Qmini VIS–NIR). Temperature was controlled by immersion of bioreactors in a water-filled glass tank fitted with heating circulator and cooling as required to maintain temperature at 35 °C ± 0.2 °C. Gas produced was collected by displacement of water in inverted 1-L measuring cylinders connected to each gas sampling port with low hydrogen-permeability tubing (Tygon E-3603, Saint-Gobain) and one-way check valves.

For long-term continuous production studies, bioreactors were fed via calibrated peristaltic pump and tubing with sterile, anaerobic nitrogen-free minimal medium containing 50 mM glycerol at a feed rate of 5 mL.h^−1^, for a dilution rate of 0.01 h^−1^. The feedstock container (10 L borosilicate reagent bottle) was connected via sterile 0.2-µm PTFE filter to a 10 L PVF gas bag filled with argon gas to avoid entrainment of oxygen or nitrogen into the anaerobic, nitrogen-free bioreactors. Concurrently, waste media was removed at equal flow rate via a liquid sampling tube and peristaltic pump to maintain constant volume in the reactor.

### *Rhodopseudomonas palustris* immobilisation

4-day cultures in VNG medium were centrifuged at 5000x*g* for 15 min in 250-mL bottles, washed twice with 100-mL aliquots of sterile phosphate buffered saline (PBS; pH 7), each followed by 5-min centrifugation. Cell pellets were weighed and resuspended in sterile 50% glycerol; bacterial biomass loading was calculated on a 1.5% wet cell weight: PVA volume basis (equating to 0.213% w/w dry cells: PVA) and the volume of 50% glycerol adjusted accordingly. 10 mL of bacterial suspension was dispersed evenly in 90 mL autoclaved 11% PVA–glycerol solution (previously melted and allowed to cool to 45 °C) for a final PVA concentration of 10%. The temperature of the mixture was held at 40 °C with stirring to maintain sufficiently low viscosity while being dripped via peristaltic pump and sterile silicone tubing into liquid nitrogen to form beads 2–3 mm in diameter. Frozen beads were immediately transferred to sterile containers and stored at −80 °C prior to use. For cultivation, beads were weighed, allowed to thaw completely at room temperature and washed twice in 500-mL aliquots of sterile PBS (pH 7) with 1-h stirring to partially remove glycerol co-solvent and unbound cells on the surface of the beads. Anaerobic incubation in excess volume nitrogen-free media (without yeast extract) overnight at 35 °C under illumination served to further remove the glycerol co-solvent without adversely affecting bacterial metabolic state and nitrogenase activity. 100 g of beads were loaded into each bioreactor with 400 mL of nitrogen-free media containing 50 mM glycerol as carbon source. Control bioreactors were inoculated with equal bacterial pellet weight as contained in the PVA beads, resuspended in nitrogen-free medium (to prevent growth and additional biomass accumulation). At each timepoint 3 mL of medium was drawn, of which 1 mL was used for biomass quantification by optical density. The remaining 2 mL was centrifuged, the cell-free supernatant decanted and frozen at −20 °C for later substrate quantification.

### Microscopic imaging

To characterise the porous properties of PVA cryogels, fluorescent staining of the material was performed by forming the cryogel as a thin layer on microscope slides. 10 µL of warm PVA solution containing ~ 1% w/w wet pellet weight *R. palustris* was spread in a thin (< 0.5 mm) even layer on a glass microscope slide and subjected to the minimum freeze–thaw cycles required to form a stable cryogel (PVA–glycerol: 1 cycle, PVA–water: 5 cycles) and washing in PBS (pH 7) at 4 °C overnight. Slides were negatively stained with 0.5 ng.mL^−1^ fluorescein in PBS (pH 9.5) for 30 min in the dark, followed by brief rinsing with deionised water to remove unpermeated stain. Imaging was performed on a Zeiss LSM 780 confocal laser scanning microscope (CLSM) using a 63x/1.4 plan apochromat objective, with z-stack optical sectioning at 10–20 µm. Fluorescein negative staining showed areas of lower relative cryogel density with a green fluorescence signal, whereas *R. palustris* autofluorescence in red (600–750 nm) was visualised with excitation from a 405-nm laser.

### Bacterial membrane integrity determination

Determination of cell membrane integrity in cultures of *R. palustris* before and after immobilisation was performed using differential fluorescent staining using propidium iodide (PI) and Syto9 (LIVE/DEAD *Bac*Light™ bacterial viability assay, ThermoFisher Scientific), with microscopic imaging by CLSM. For pre-immobilisation samples, 1 mL of a 3-day culture grown in VNG medium was centrifuged (6500x*g*, 5 min) and resuspended in PBS. 100-µL aliquots of cell suspension were stained with addition of PI and Syto9 to final concentrations of 30 and 0.334 µM, respectively, followed by 10-min incubation in the dark prior to imaging. Beads of PVA cryogel-entrapped cultures were collected after cultivation, rinsed in PBS and cut in half, followed by incubation in 2X staining solution (60 µM PI, 0.668 µM Syto9 in PBS) for 10 min in the dark. Beads were placed cut-side down in an 8-well chambered coverglass for imaging (Nunc Lab-Tek™, ThermoFisher Scientific). A minimum of 3 beads/samples were imaged for each timepoint, with 4 randomly chosen fields of view each (which did not contain non-bacterial debris). 6-layer z-stacks were acquired for each field of view, at a depth of 1.3 µm per layer. For image analysis, sequential pairs of z-stack layers were exported as single maximum intensity orthogonal projections, with identical channel intensity settings for all images.

Cell counting was performed using the BioFilmAnalyzer tool [65]. Settings for cell size range and threshold were determined by closely matching output to the values from manual counts performed on 4 sample images. Viability was assessed as the proportion of membrane-intact cells to total cells (intact and compromised), expressed as percentage.

### Analytical

Culture growth was quantified by optical density at 660 nm and correlated to dry cell weight using a standard curve. Glutamate concentration was determined by colorimetric ninhydrin assay [66], modified by increasing ethanol concentration to minimise the effect of sample pH and to both enhance and stabilise chromophore formation. Briefly, 400 µL of 4 mM ninhydrin in absolute ethanol was added to 100 µL of 0.2 µm-filtered sample in 1.5 mL microfuge tubes and incubated in a water bath at 80 °C for 15 min. 200 µL of each reaction, including standards, was pipetted in duplicate into a wells of a flat-bottom 96-well plate (Greiner) and absorbance read at 590 nm in a microplate reader (ELx800, BioTek instruments) within 30 min.

Glycerol was quantified by HPLC, using a Dionex UltiMate 3000 system fitted with a Biorad HPX-87H 250 x 7.8 mm column with guard cartridge and ERC Refracto Max520 refractive index detector. 20 µL of sample was injected and eluted with 0.005 M H_2_SO_4_ mobile phase at 0.6 mL/min and column temperature of 65 °C.

A Global Analyser Solutions CompactGC with argon carrier stream and thermal conductivity detector was employed to confirm the relative composition of the gas produced. H_2_ and CO_2_ were quantified and reported as relative percentages; any contaminating nitrogen or oxygen was discounted as artefact of the sampling procedure. Typical gas composition fell within the range of 94.1 ± 0.4% H_2_, 5.9 ± 0.4% CO_2_.

Data analysis was performed in GraphPad Prism 7. One- or two-way ANOVA followed by Tukey’s or Sidak’s multiple comparisons tests, respectively, was used to determine statistical significance at *p* < 0.05. Significance level is indicated by ****p < 0.0001; ***p < 0.001; **p < 0.01; *p < 0.05; *ns* not significant. Error bars indicate SD or SEM and sample size (n) is given for each figure.

## Supplementary information

**Additional file 1:** Supplementary methods and sample results for determination of diffusion coefficients in PVA cryogels.

## Data Availability

All data and material used in the current study are available from the corresponding author on reasonable request.
