# Supplementary material for: Transparent polyvinyl-alcohol cryogel as immobilisation matrix for continuous biohydrogen production by phototrophic bacteria
Source: Biotechnol Biofuels. 2020 Jun 9;13:105. doi: 10.1186/s13068-020-01743-7 (PMC7285740; doi:10.1186/s13068-020-01743-7)
Supplement: Supplementary file 1 — Additional file 1: Supplementary methods and sample results for determination of diffusion coefficients in PVA cryogels. [file 13068_2020_1743_MOESM1_ESM.pdf]

Transparent poly vinyl-alcohol cryogel as immobilization matrix for continuous biohydrogen production by phototrophic bacteria

Jan-Pierre du Toit and Robert W. M. Pott

Supplementary methods

In order to determine the effective diffusivity D_{eff}, the partition coefficient defined according to the equilibrium relationship between substrate contained in the PVA cryogel cube (c_{cube}) and the solution (c_{sol}) such that $c_{sol} = K_H c_{cube}$, must first be determined. Cubes were equilibrated with a solution of known substrate concentration ($c_{sol,eq}$) and subsequently placed in a substrate free solution ($c_{sol} = 0$ at $t = 0$) until a new equilibrium was reached such that $\lim_{t\to\infty} c_{sol} = c_{sol,f}$, as described in the experimental methods section. The equilibrium relationship with the initial solution (eq. 1), final solution (eq. 2) and a mass balance over the diffusion experiment (eq. 3) yields a system of linear equations which can be readily solved for the unknown values K_H, $c_{cube,0}$ and $c_{cube,f}$:

$$c_{sol,eq} = K_H c_{cube,0} \tag{1}$$

$$c_{sol,f} = K_H c_{cube,f} \tag{2}$$

$$V_{sol} c_{sol,f} = V_{cube}\left(c_{cube,0} - c_{cube,f}\right) \tag{3}$$

Where V_{sol} and V_{cube} are the liquid solution and cube volumes, respectively. Once the initial cube concentration $c_{cube,0}$ and the partition coefficient K_H are known, the intra-cube substrate diffusion can be modelled by approximating the cube as a sphere with radius equal to the surface-volume radius of the cube ($R = 3V_{cube}/A_{cube}$ where A_{cube} is the surface area of the cube). The partial differential equation describing the diffusion process is given by eq. 4, with accompanying boundary conditions (eqns. 5-6) and initial condition (eq. 7):

$$\frac{\partial c}{\partial t} = \mathcal{D}_{eff}\left(\frac{\partial c}{\partial r^2} + \frac{2}{r}\frac{\partial c}{\partial r}\right) \quad \text{for } 0 < r < R, t > 0 \tag{4}$$

$$\frac{\partial c}{\partial r} = 0 \quad \text{for } r = 0, t > 0 \tag{5}$$

$$-\mathcal{D}_{eff}\frac{\partial c}{\partial r} = k_L(K_H c - c_{sol}) \quad \text{for } r = R, t > 0 \tag{6}$$

$$c = c_{cube,0} \quad \text{for } 0 \leq r \leq R, t = 0 \tag{7}$$

Equation 6 represents the flux balance at the surface of the cube, where the flux out of the cube is equal to the rate of mass transfer from the surface of the cube to the bulk solution, with mass transfer coefficient k_L. The concentration of the bulk solution is modelled using the ordinary differential equation (ODE) given by eq. 8, with the initial condition $c_{sol}(0) = 0$:

$$V_{sol}\frac{dc_{sol}}{dt} = k_L A_{cube}(K_H c - c_{sol}) \tag{8}$$

The partial differential equation (eq. 4) was discretized using the finite difference method and combined with eq. 8, yielding a system of ODEs. This system of ODEs is readily solved using MATLAB (Natick, MA) and the built-in numerical integrator ode45.

The substrate concentration was measured at time points t_i during the diffusion experiment, yielding the experimental measurements $\hat{c}_{sol}(t_i)$. The unknown parameters $\mathcal{D}_{eff}$ and k_L was determined by regressing the model predicted values $c_{sol}(t_i)$ against the experimental measurements $\hat{c}_{sol}(t_i)$. The regression was performed using the MATLAB built-in least-squares optimization function lsqnonlin.

Results

The regression results showed that the effective diffusivity D_{eff} was insensitive to k_L when k_L > 10^{-3}. In fact, a basic identifiability analysis indicated that k_L was practically unidentifiable. This is illustrated in Figure X1 for transparent cryogels with glycerol substrate. The optimal effective diffusivity was determined to be D_{eff} = 7.32 x 10^{-6} $cm^2.s^{-1}$. Model results compared to experimental measurements are shown in Figure X2, indicating an excellent fit.

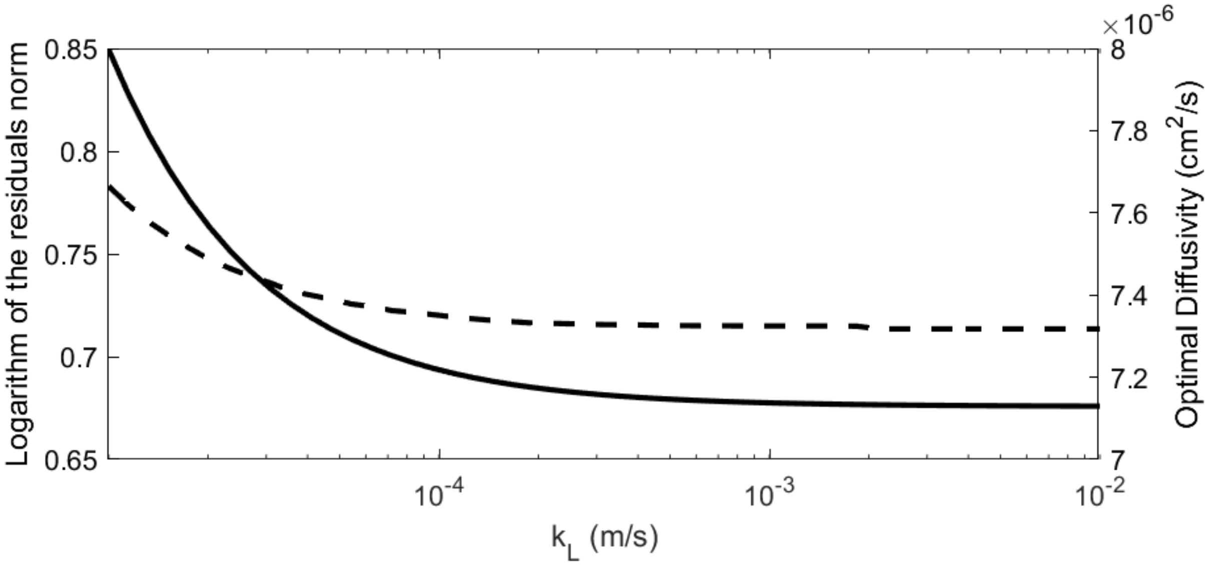

Figure X1: The minimum residual error (solid line) decreases asymptotically as the mass transfer coefficient k_L increases, indicating practical unidentifiability. The optimal diffusivity D_{eff} (dashed line) is insensitive to variations in k_L above a value of 10^{-3} and tends to a value of $\mathcal{D}_{eff}$ = 7.32 x 10^{-6} $cm^2.s^{-1}$.

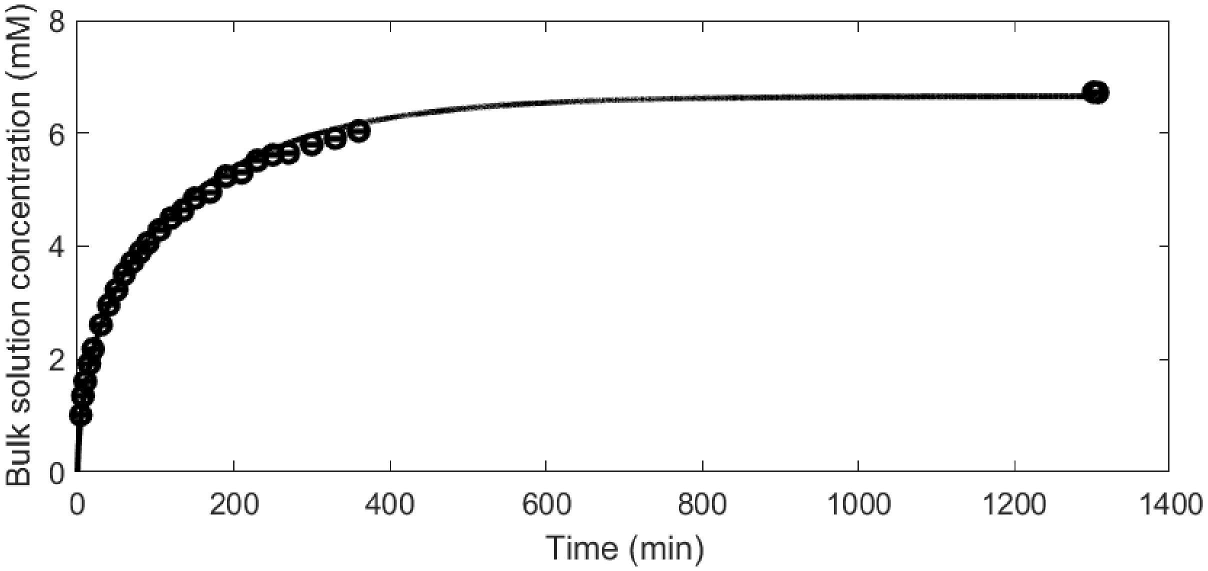

Figure X2: Model predictions (solid line) using $\mathcal{D}_{eff}$ = 7.32 x 10^{-6} $cm^2.s^{-1}$, compared to experimental measurements (circles) for diffusion of glycerol from transparent cryogel.
